# Premorbid cancer and motor reserve in patients with Parkinson’s disease

**DOI:** 10.1038/s41598-022-13322-x

**Published:** 2022-06-03

**Authors:** Yoon-Sang Oh, Sang-Won Yoo, Chul Hyoung Lyoo, Kwang-Soo Lee, Joong-Seok Kim

**Affiliations:** 1 Department of Neurology, College of Medicine, The Catholic University of Korea, Seoul, Republic of Korea; 2 Department of Neurology, Gangnam Severance Hospital, Yonsei University College of Medicine, Seoul, Republic of Korea; 3 Department of Neurology, Seoul St. Mary′s Hospital, College of Medicine, The Catholic University of Korea, 222, Banpo-daero, Seocho-gu, Seoul, 06591 Republic of Korea

**Keywords:** Neurology, Neurological disorders

## Abstract

Decreased cancer risk has been reported in patients with Parkinson’s disease (PD), and cancer prior to PD can have a protective effect on PD risk. We investigated cancer history prior to PD diagnosis to determine if such history can enhance motor reserve in PD by assessing the association between motor deficits and striatal subregional dopamine depletion. A total of 428 newly diagnosed, drug-naïve PD patients was included in the study. PD patients were categorized into three groups of no prior neoplasia, premorbid precancerous condition, and premorbid malignant cancer before PD diagnosis. Parkinsonian motor status was assessed using the Unified Parkinson’s Disease Rating Scale (UPDRS) motor score and modified Hoehn and Yahr stage score. All patients underwent positron emission tomography (PET) with ^18^F-N-(3-fluoropropyl)-2beta-carbon ethoxy-3beta-(4-iodophenyl) nortropane (^18^F-FP-CIT), and the regional standardized uptake value ratios (SUVRs) were analyzed with a volume-of-interest template among the groups. The UPDRS motor score negatively correlated with SUVRs in the posterior putamen for all patient groups. Groups with neoplasia, especially those with premorbid cancer, showed lower motor scores despite similar levels of dopamine depletion in the posterior putamen relative to those without neoplasia. These results suggest that premorbid cancer acts as a surrogate for motor reserve in patients with PD and provide imaging evidence that history of cancer has a protective effect on PD.

## Introduction

Emerging evidence suggests that patients with Parkinson’s disease (PD) have a low risk of various types of cancers compared to the general population^1^. A nationwide study in the United Kingdom showed a reduced risk for development of cancer in PD patients^2^, and a recent population-based cohort study showed that patients with PD had a lower overall cancer risk^3^. A recent meta-analysis study revealed a similar result^4^. PD has been shown to have reduced risks of smoking-related and several non-smoking-related cancers^5^, and a decreased incidence of most cancers, except malignant melanoma, was found in patients with PD^6^. Patients with any cancer prior to PD diagnosis have a lower risk of developing PD, which indicates that cancer might have a protective effect on PD^7^. However, one East Asian study demonstrated conflicting results that cancer incidence was increased in patients with PD^8^.

PD and cancer have similar genetic backgrounds, and genetic factors that protect against cancer also are linked to the neurodegeneration of PD^9^. Mutations in genes linked to PD and/or cancer involve changes in cell cycle control, which influence susceptibility to disease^9^. Cancer and PD involve changes in similar cellular pathways and PD might have some biological protection against cancer^10^.

PD is mainly characterized by motor dysfunction and non-motor symptoms associated with nigrostriatal dopaminergic depletion. Recently, the concept of “motor reserve,” which explains the individual deficits despite similar pathological changes in the nigrostriatal pathway in PD^11^, has been associated with reduced motor deficits despite severe pathological changes. The degree of motor reserve can be represented by motor score of Unified Parkinson’s disease rating scale (UPDRS) and the level of striatal dopamine depletion. Patients with high motor reserve exhibited fewer motor deficits i.e., lower UPDRS motor score, compared with those with low motor reserve despite similar levels of dopamine transporter activity in the striatum, especially in the posterior putamen^11^.

Epidemiological evidences suggested that if cancer is protective in PD, we presumed that cancer would affect dopamine of nigrostriatal pathway and/or might prevent nigrostriatal cell degeneration in PD. Therefore, we hypothesized that premorbid cancer in PD is expected to have a positive influence on the “motor reserve” of the nigrostriatal pathway in the pathophysiology of PD. Herein, we investigated the difference between striatal dopamine activity and severity of motor deficit in the relationship between PD and neoplasia in a variety of clinical settings.

## Results

**Figure 1:**
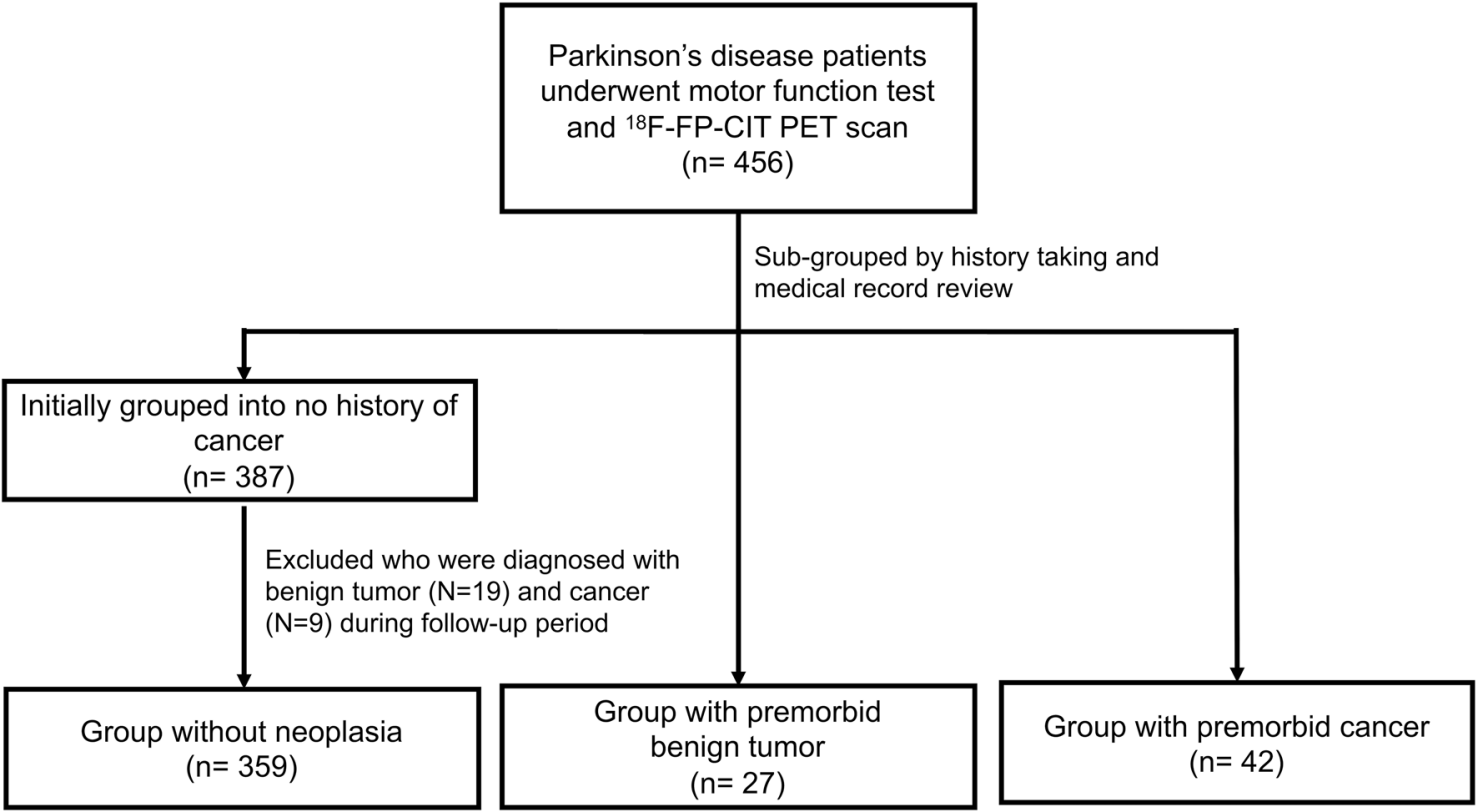
Enrolled patient study flow.

The patient flow chart is shown in Fig. 1. A total of 456 patients diagnosed with PD was enrolled initially in this study. Among them, 28 patients were excluded because of benign tumors/malignant cancers during follow-up from the time of PD diagnosis to the time of the study. Finally, 359 patients with no history of neoplasia, 27 with premorbid benign tumor, and 42 with premorbid cancer were included in this study.

The mean age was 71.5 ± 9.5 years, and 220 (51.4%) patients were male. The mean disease duration was 1.2 ± 1.2 years. A total of 209 (48.8%) patients had hypertension, and 95 patients (22.2%) had diabetes mellitus. The number of non-smokers was 334 (78.0%), ex-smokers was 77 (18.0%), and current smokers was 17 (4.0%). Mean UPDRS total score was 25.3 ± 13.5 (UPDRS part I: 2.4 ± 2.1, UPDRS part II: 7.2 ± 4.8, and UPDRS part III: 15.7 ± 8.8), and the median modified H&Y stage score was 2.0 (interquartile range, 1.0). The baseline clinical characteristics of each group are summarized in Table 1. The most common benign tumors were gastrointestinal adenomatous polyps, and the most common cancers were colorectal and thyroid cancer. In the premorbid cancer group, there were carcinoma in 40 patients, sarcoma in one patient, and one in lymphoma based on histopathological classification. No melanoma patients were identified. The average duration of cancer prevalence before PD diagnosis was 7.4 ± 3.4 years, and the mean duration of benign tumors was 5.8 ± 2.9 years. The prior neoplasia group had lower UPDRS total and part III scores than those without neoplasia.

**Table 1:** Baseline clinical characteristics.

	Group with no prior neoplasia (n = 359)^a^	Group with prior benign tumor (n = 27)^b^	Group with prior cancer (n = 42)^c^	*P*	Post-hoc analysis
Age, yr	71.1 ± 9.7	71.9 ± 10.5	74.0 ± 6.9	0.165	–
Sex, male, n, %	180 (50.1%)	16 (59.3%)	24 (57.1%)	0.484	–
Disease duration, yr	1.1 ± 1.2	1.5 ± 1.6	1.1 ± 1.2	0.338	–
Hypertension, n, %	172 (47.9%)	14 (51.9%)	23 (54.8%)	0.666	–
Diabetes mellitus, n, %	75 (20.9%)	6 (22.2%)	14 (33.3%)	0.185	–
**Smoking status**				0.251	–
Non-smoker, n, %	286 (79.7%)	17 (63.0%)	31 (73.8%)		–
Ex-smoker, n, %	59 (16.4%)	9 (33.3%)	9 (21.4%)		–
Current smoker, n, %	14 (3.9%)	1 (3.7%)	2 (4.8%)		–
Tumor/cancer classification	–	Colorectal adenomatous polyp (10), gastric adenomatous polyp (4), renal cyst (3), benign thyroid nodule (4), pulmonary nodule (2), hepatic cystic nodule (1), adrenal tumor (1), Schwanoma of forearm (1), breast cyst (1)	Thyroid cancer (9), colorectal cancer (8), gastric cancer (6), lung cancer (5), breast cancer (4), prostate cancer (2), bladder cancer (2), renal cell carcinoma (2), lymphoma (1), basal cell carcinoma of skin (1), cervical cancer (1), retroperitoneal sarcoma (1)		
UPDRS	26.0 ± 13.9	22.8 ± 11.6	20.9 ± 10.9	0.039	a > c
UPDRS Part I	2.4 ± 2.2	1.9 ± 1.9	2.0 ± 1.9	0.242	–
UPDRS Part II	7.5 ± 4.9	6.1 ± 3.2	6.1 ± 4.4	0.101	–
UPDRS Part III	16.1 ± 8.8	14.7 ± 9.1	12.8 ± 7.5	0.053	a > c
H&Y, median (IQR)	2.0 (1.0)	2.0 (1.0)	2.0 (1.0)	0.697	–

Values represent mean with standard deviation, numbers of patients (percentage) or median with interquartile range (IQR).

Group differences were compared with one-way analysis of variance with least significant difference post-hoc test, Pearson's χ^2^ test or Kruskal–Wallis test, as appropriate.

*UPDRS* Unified Parkinson’s Disease Rating Scale, *H*&*Y* Hoehn and Yahr stage score.

The SUVRs of ^18^F-FP-CIT PET imaging were analyzed. The SUVRs of patient groups showed significant reductions in whole striatal subregions than those of controls, however, there were no significant differences among patient groups (Table 2). The non-parametric Spearman’s correlation analysis indicated that UPDRS part III score was more negatively correlated with SUVRs in the caudate (*r* = − 0.296, *p* < 0.001), putamen (*r* = − 0.312, *p* < 0.001), globus pallidus (*r* = − 0.110, *p* = 0.023), thalamus (*r* = − 0.239, *p* < 0.001), and ventral striatum (*r* = − 0.297, *p* < 0.001). This correlation continued in the posterior putamen (*r* = − 0.292, *p* < 0.001). In the groups with prior neoplasia, UPDRS part III score was also more negatively correlated with SUVRs than those without neoplasia; in the putamen (group with malignant cancer vs. group with benign tumor vs. group without neoplasia; *r* = − 0.602 vs. *r* = − 0.435 vs. *r* = − 0.267), globus pallidus (*r* = − 0.382 vs. *r* = − 0.244 vs. *r* = − 0.059), thalamus (*r* = − 0.325 vs. *r* = − 0.085 vs. *r* = − 0.238), and ventral striatum (*r* = − 0.621 vs. *r* = − 0.319 vs. *r* = − 0.266), respectively. The correlation was higher in the posterior putamen (group with malignant cancer; *r* = − 0.510, group with benign tumor; *r* = − 0.416) than in those without neoplasia (*r* = − 0.250).

**Table 2:** Comparison of striatal dopamine activities among groups.

Striatal subregion	Group with no prior neoplasia (n = 359)^a^	Group with prior benign tumor (n = 27)^b^	Group with prior cancer (n = 42)^c^	Normal controls (n = 22)^d^	*P*	Post hoc analysis
Caudate	3.62 ± 1.41	3.40 ± 1.42	3.53 ± 1.41	5.80 ± 1.12	< 0.001	a = b = c < d
Anterior	3.80 ± 1.60	3.56 ± 1.64	3.69 ± 1.63	6.21 ± 1.33	< 0.001	a = b = c < d
Posterior	2.76 ± 1.17	2.51 ± 1.01	2.79 ± 1.11	4.69 ± 0.87	< 0.001	a = b = c < d
Putamen	4.06 ± 1.08	4.10 ± 1.03	4.27 ± 1.22	7.95 ± 0.97	< 0.001	a = b = c < d
Anterior	4.12 ± 1.23	4.21 ± 1.26	4.42 ± 1.40	8.39 ± 1.22	< 0.001	a = b = c < d
Posterior	3.16 ± 1.00	3.22 ± 1.04	3.44 ± 1.23	7.91 ± 0.91	< 0.001	a = b = c < d
Ventral	3.85 ± 0.83	3.76 ± 0.94	3.80 ± 0.89	6.03 ± 0.86	< 0.001	a = b = c < d
Globus pallidus	3.43 ± 0.95	3.51 ± 0.86	3.70 ± 1.10	5.03 ± 0.98	< 0.001	a = b = c < d
Thalamus	1.44 ± 0.17	1.44 ± 0.18	1.45 ± 0.18	1.63 ± 0.12	< 0.001	a = b = c < d
Ventral striatum	4.98 ± 1.28	4.92 ± 1.18	4.91 ± 1.24	7.14 ± 1.57	< 0.001	a = b = c < d

Values represent mean with standard deviation.

Analyses were performed using analysis of covariance (ANCOVA) with least significance difference post hoc test after controlling for age.

**Figure 2:**
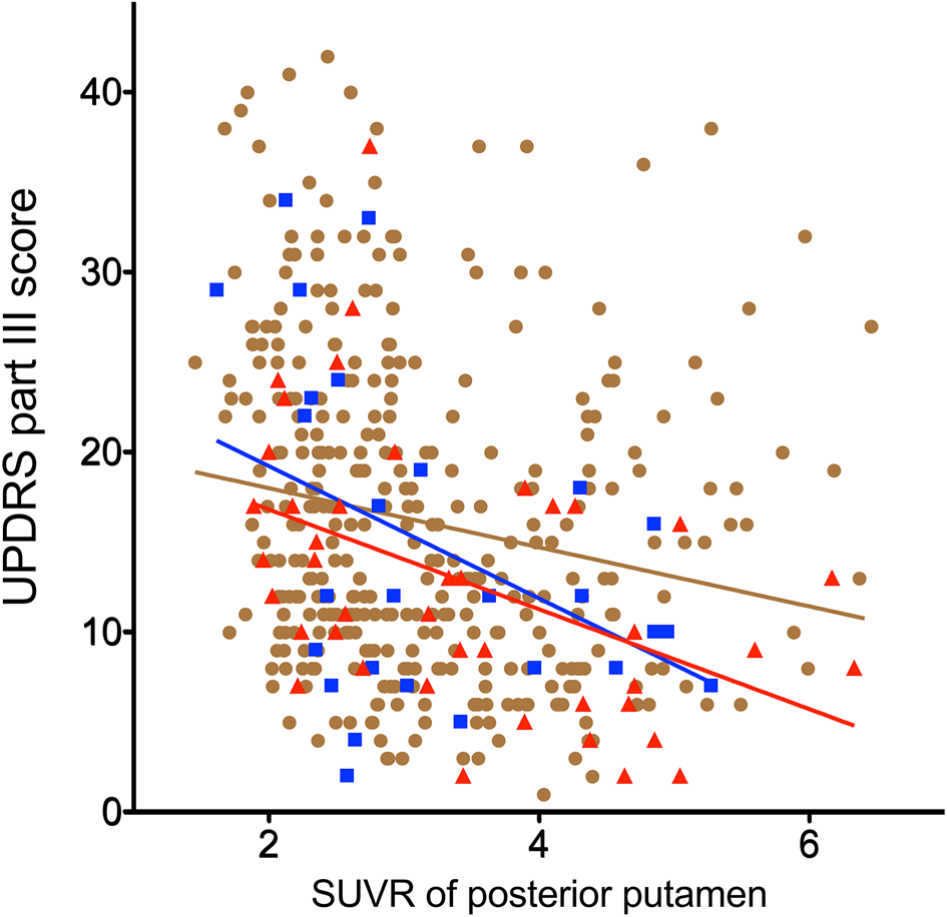
Scatter plot showing the relationship between dopamine transporter activity represented by standardized uptake value ratios (SUVRs) and motor deficits (UPDRS part III score) among groups. The premorbid malignant cancer group (red color) showed lower UPDRS part III scores at a similar level of SUVRs of the posterior putamen compared to the no prior neoplasia group (brown color). The premorbid precancerous condition group (blue color) exhibited a significantly steeper slope than those without neoplasia (brown color). The calculated formulae of the relationship from a general linear model were as follows: no prior neoplasia group, UPDRS part III = 21.31 − 1.64 × SUVR of posterior putamen; premorbid precancerous condition group, UPDRS part III = 26.56 − 3.68 × SUVR of posterior putamen; premorbid malignant cancer group, UPDRS part III = 22.35 − 2.78 × SUVR of posterior putamen.

The differences of Pearson’s correlation coefficient between UPDRS score and striatal subregional dopamine activities were determined within groups (Table 3). The correlation coefficients between putamen and UPDRS part III were different between the no prior neoplasia group and that of prior cancer before PD; the premorbid malignant cancer group exhibited lower UPDRS part III scores at a similar level of SUVRs of the posterior putamen than did those with no prior neoplasia. This significance was continued in the premorbid carcinoma subgroup among the premorbid cancer group (Supplementary Tables 1 and 2). The ratios of SUVR of posterior putamen/UPDRS part III score were also significant higher in premorbid cancer group than group without neoplasia (group with malignant cancer vs. group with benign tumor vs. group without neoplasia: 0.48 ± 0.56 vs. 0.35 ± 0.28 vs. 0.30 ± 0.31, *p* = 0.001; Table 4). Subjects with premorbid precancerous conditions had a steeper slope than those without neoplasia (Fig. 2). Comparison between premorbid precancerous condition and premorbid malignant cancer groups did not show differences in correlation coefficients between the SUVRs of the posterior putamen and UPDRS part III score.

**Table 3:** Comparisons of correlation coefficient within group.

	No neoplasia (group 1) versus benign tumor (group 2)				No neoplasia (group 1) versus cancer (group 3)				Benign tumor (group 2) versus cancer (group 3)			
	Z1	Z2	Z	*P*	Z1	Z3	Z	*P*	Z2	Z3	Z	*P*
**UPDRS part 1**												
Caudate	− 0.250	− 0.465	1.018	0.154	− 0.250	− 0.364	0.677	0.249	− 0.465	− 0.364	− 0.387	0.349
Anterior	− 0.249	− 0.442	0.913	0.181	− 0.249	− 0.367	0.679	0.243	− 0.442	− 0.367	− 0.289	0.386
Posterior	− 0.249	− 0.457	0.988	0.162	− 0.249	− 0.455	1.221	0.111	− 0.457	− 0.455	− 0.009	0.496
Putamen	0.033	− 0.123	0.738	0.230	0.033	0.078	− 0.268	0.394	− 0.123	0.078	− 0.774	0.220
Anterior	0.013	− 0.144	0.744	0.228	0.013	0.028	− 0.030	0.488	− 0.144	0.028	− 0.663	0.254
Posterior	0.111	0.203	− 0.433	0.333	0.111	0.153	− 0.247	0.402	0.203	0.153	0.191	0.424
Ventral	− 0.007	− 0.109	0.486	0.314	− 0.007	0.025	− 0.190	0.425	− 0.109	0.025	− 0.518	0.302
Globus pallidus	0.234	− 0.008	1.148	0.125	0.234	0.096	0.818	0.207	− 0.008	0.096	− 0.402	0.344
Thalamus	− 0.157	− 0.373	1.025	0.153	− 0.157	− 0.246	0.525	0.300	− 0.373	− 0.246	− 0.492	0.311
Ventral striatum	− 0.095	− 0.555	2.178	0.015*	− 0.095	− 0.044	− 0.304	0.381	− 0.555	− 0.044	− 1.968	0.025*
**UPDRS part 2**												
Caudate	− 0.353	− 0.305	− 0.227	0.410	− 0.353	− 0.418	0.384	0.351	− 0.305	− 0.418	0.434	0.332
Anterior	− 0.356	− 0.295	− 0.290	0.386	− 0.356	− 0.398	0.245	0.403	− 0.295	− 0.398	0.395	0.346
Posterior	− 0.307	− 0.280	− 0.129	0.449	− 0.307	− 0.457	0.890	0.187	− 0.280	− 0.457	0.684	0.247
Putamen	− 0.226	− 0.221	− 0.025	0.490	− 0.226	− 0.385	0.944	0.173	− 0.221	− 0.385	0.634	0.263
Anterior	− 0.245	− 0.195	− 0.234	0.408	− 0.245	− 0.377	0.783	0.217	− 0.195	− 0.377	0.699	0.242
Posterior	− 0.192	− 0.096	− 0.455	0.324	− 0.192	− 0.352	0.946	0.172	− 0.096	− 0.352	0.985	0.162
Ventral	− 0.206	− 0.368	0.768	0.221	− 0.206	− 0.354	0.879	0.190	− 0.368	− 0.354	− 0.053	0.479
Globus pallidus	0.047	− 0.130	0.838	0.201	0.047	− 0.160	1.230	0.109	− 0.130	− 0.160	0.118	0.453
Thalamus	− 0.201	− 0.251	0.239	0.405	− 0.201	− 0.317	0.691	0.245	− 0.251	− 0.317	0.255	0.399
Ventral striatum	− 0.246	− 0.346	0.476	0.317	− 0.246	− 0.390	0.852	0.197	− 0.346	− 0.390	0.167	0.434
**UPDRS part 3**												
Caudate	− 0.335	− 0.199	− 0.647	0.259	− 0.335	− 0.357	0.133	0.447	− 0.199	− 0.357	0.613	0.270
Anterior	− 0.338	− 0.187	− 0.717	0.237	− 0.338	− 0.338	0.000	0.500	− 0.187	− 0.338	0.583	0.280
Posterior	− 0.268	− 0.140	− 0.609	0.271	− 0.268	− 0.212	− 0.333	0.370	− 0.140	− 0.212	0.278	0.390
Putamen	− 0.231	− 0.510	1.323	0.093	− 0.231	− 0.623	2.322	0.010*	− 0.510	− 0.623	0.434	0.332
Anterior	− 0.236	− 0.476	1.136	0.128	− 0.236	− 0.599	2.148	0.016*	− 0.476	− 0.599	0.472	0.318
Posterior	− 0.188	− 0.446	1.225	0.110	− 0.188	− 0.488	1.780	0.038*	− 0.446	− 0.488	0.162	0.436
Ventral	− 0.194	− 0.451	1.218	0.112	− 0.194	− 0.613	2.480	0.007**	− 0.451	− 0.613	0.622	0.267
Globus pallidus	0.012	− 0.364	1.784	0.037*	0.012	− 0.266	1.649	0.050	− 0.364	− 0.266	− 0.378	0.353
Thalamus	− 0.270	− 0.066	− 0.969	0.166	− 0.270	− 0.313	0.251	0.401	− 0.066	− 0.313	0.951	0.171
Ventral striatum	− 0.277	− 0.387	0.523	0.300	− 0.277	− 0.698	2.496	0.006**	− 0.387	− 0.698	1.197	0.116
**UPDRS total**												
Caudate	− 0.381	− 0.318	− 0.299	0.382	− 0.381	− 0.494	0.664	0.253	− 0.318	− 0.494	0.675	0.250
Anterior	− 0.385	− 0.302	− 0.394	0.347	− 0.385	− 0.470	0.503	0.308	− 0.302	− 0.470	0.647	0.259
Posterior	− 0.322	− 0.262	− 0.284	0.388	− 0.322	− 0.413	0.541	0.294	− 0.262	− 0.413	0.583	0.280
Putamen	− 0.222	− 0.482	1.236	0.108	− 0.222	− 0.574	2.087	0.018*	− 0.482	− 0.574	0.352	0.362
Anterior	− 0.235	− 0.453	1.030	0.151	− 0.235	− 0.565	1.958	0.025*	− 0.453	− 0.565	0.435	0.332
Posterior	− 0.171	− 0.333	0.769	0.221	− 0.171	− 0.454	1.679	0.047*	− 0.333	− 0.454	0.466	0.320
Ventral	− 0.198	− 0.481	1.344	0.089	− 0.198	− 0.564	2.173	0.015*	− 0.481	− 0.564	0.320	0.374
Globus pallidus	0.060	− 0.321	1.805	0.036*	0.060	− 0.232	1.732	0.042*	− 0.321	− 0.232	− 0.341	0.367
Thalamus	− 0.269	− 0.181	− 0.419	0.338	− 0.269	− 0.394	0.740	0.230	− 0.181	− 0.394	0.822	0.206
Ventral striatum	− 0.279	− 0.504	1.065	0.143	− 0.279	− 0.655	2.229	0.013*	− 0.504	− 0.655	0.583	0.280

Analyses were performed using Pearson’s z test as following formula.

$$\begin{array}{*{20}l} {{\text{Z}}_{{\text{a}}} \, = \,0.{5}[{\text{ln}}\left( {{1}\, + \,r_{{\text{a}}} } \right){-}\left( {{\text{ln}}\left( {{1}\, + \,r_{{\text{a}}} } \right)} \right]} \hfill \\ {{\text{Z}}_{{\text{b}}} \, = \,0.{5}[{\text{ln}}\left( {{1}\, + \,r_{{\text{b}}} } \right){-}\left( {{\text{ln}}\left( {{1}\, + \,r_{{\text{b}}} } \right)} \right]} \hfill \\ \end{array} \quad \sigma_{{Z_{a} - Z_{b } }} = \sqrt {\frac{1}{{n_{a} - 3}} + \frac{1}{{n_{b} - 3}}} \quad Z = \frac{{Z_{a} - Z_{b} }}{{\sigma_{{Z_{a} - Z_{b} }} }}$$.

*< 0.05, **< 0.01.

**Table 4:** Ratios of striatal dopamine activities/UPDRS part III score.

Striatal subregion	Group with no prior neoplasia (n = 359)^a^	Group with prior benign tumor (n = 27)^b^	Group with prior cancer (n = 42)^c^	*P*	Post hoc analysis
Caudate	0.35 ± 0.35	0.38 ± 0.36	0.52 ± 0.76	0.004	c > a
Anterior	0.37 ± 0.37	0.39 ± 0.38	0.55 ± 0.84	0.004	c > a
Posterior	0.26 ± 0.26	0.28 ± 0.27	0.39 ± 0.51	0.002	c > a
Putamen	0.38 ± 0.41	0.45 ± 0.37	0.61 ± 0.73	0.003	c > a
Anterior	0.39 ± 0.43	0.46 ± 0.37	0.64 ± 0.83	0.002	c > a
Posterior	0.30 ± 0.31	0.35 ± 0.28	0.48 ± 0.56	0.001	c > a
Ventral	0.36 ± 0.35	0.42 ± 0.38	0.53 ± 0.62	0.006	c > a
Globus pallidus	0.31 ± 0.36	0.38 ± 0.35	0.48 ± 0.48	0.010	c > a
Thalamus	0.13 ± 0.12	0.15 ± 0.14	0.19 ± 0.19	0.008	c > a
Ventral striatum	0.47 ± 0.48	0.54 ± 0.47	0.70 ± 0.88	0.008	c > a

Values represent mean with standard deviation.

Analyses were performed using analysis of covariance (ANCOVA) with Bonferroni post hoc test after controlling for age.

The general linear model was performed to investigate differences between UPDRS part III score and SUVRs of the posterior putamen (Table 5). The premorbid malignant cancer group had lower estimates of UPDRS part III score than those without neoplasia after adjusting for covariates (*p* = 0.009).

**Table 5:** UPDRS motor score estimates within groups.

	Unadjusted		Adjusted^a^	
	B (S.E.)	*P*	B (S.E.)	*P*
SUVRs of posterior putamen	− 1.925 (0.402)	< 0.001	− 2.167 (0.398)	< 0.001
No neoplasia	Reference		Reference	
Premorbid benign tumor	− 1.292 (1.697)	0.447	− 1.518 (1.648)	0.357
Premorbid cancer	− 2.785 (1.391)	0.046	− 3.561 (1.356)	0.009

Analyses were performed by general linear model.

*B* estimated difference, *S.E.* standard error, *SUVRs* standardized uptake value ratios.

^a^Adjusted for age, sex, symptom duration, hypertension, diabetes mellitus and smoking status.

## Discussion

This study demonstrates that patients with cancer prior to PD diagnosis have a different correlation between posterior putamen dopamine depletion and motor deficits compared to those without neoplasia. Patients with prior cancer exhibited lower UPDRS scores compared to those without neoplasia despite having similar dopamine transporter activity in the posterior putamen. This result suggests that premorbid cancer prior to PD can preserve initial motor reserve, which reflects individual capacity to tolerate PD pathology. Therefore, this study suggests that premorbid cancer has a protective effect on striatal dopamine in PD patients.

PD and cancer have similar genetic backgrounds. A specific genetic factor that can protect from cancer also can have a predisposal effect on neurodegeneration in PD^9^. As PD-linked genes have been found in various cancers, mutations in PD genes might have a common pathway to cancer pathogenesis that is associated with development of cancer^9^. Cell cycle dysregulation of apoptosis is a main mechanism of PD and cancer. The ubiquitin–proteasome system (UPS) is important for cell cycle control, protects against disease pathogenesis, and causes cell death^9^. Alteration in the UPS pathway and normal cell cycle can increase susceptibility to disease; however, alteration of UPS function is different between the two diseases^9^. Cancer has been associated with UPS overexpression, while PD has been associated with inhibition of UPS function^12^. Therefore, PD patients with premorbid cancer have a decreased tendency of cell apoptosis-associated dopaminergic neuronal loss. A recent study revealed that patients with skin cancer or any cancer before PD diagnosis had low risk for PD^7^. In our study, only one patient had skin cancer, and the other patients had 11 separate types of cancer; therefore, we suggest that any cancer prior to PD diagnosis has a protective effect against striatal dopamine depletion.

Benign tumors are a classical nosology. At the outset, lesions produced by carcinogens are not cancerous but are focal proliferations that are orderly in form and restricted in growth. Some benign tumors are regarded as precancerous lesions on the basis of growth characteristics, temporally restricted (non-autonomous) or temporally unrestricted (autonomous or semi-autonomous), and whether lesional growth is confined to a single tissue compartment or involves two or more tissue compartments^13^. These benign lesions including colorectal adenomatous polyp^14^, gastric adenomatous polyp^15^, renal cyst^16^, thyroid nodule^17^, pulmonary nodule^18^, hepatic cyst^19^, adrenal tumor^20^, and/or breast cyst^21^ impart increased risk of developing cancer. In many cases, benign tumors have the potential to become malignant through a process known as tumor progression^13^. It is thought that cancer is preceded by a clinically silent premalignant phase during which oncogenic genetic and epigenetic alterations accumulate^22^. In this study, individuals with benign tumor before PD diagnosis had a minor association between striatal dopamine activity, especially of the globus pallidus, and motor deficit. Therefore, we postulate that precancerous benign tumor can influence the dopaminergic system through a variety of oncogenic genetic and epigenetic alterations, although more studies are needed.

This study has several strengths. First, we only enrolled patients with newly diagnosed PD who had taken no anti-parkinsonian medication. Second, there were no melanoma patients in our study. Unlike Western people, melanoma is relatively rare in Asian people. While PD apparently offers protection against certain cancers, many studies have reported a high risk of melanoma in PD patients and co-occurrence of PD in melanoma patients and melanoma in PD patients^23^. Therefore, inclusion of melanoma subjects can bias the result of this study. Third, we excluded patients who were diagnosed with neoplasia after PD diagnosis. Many cancer/precancerous lesions have a variety of genetic predispositions; therefore, we speculated that later occurrence of cancer/benign tumors could bias nigrostriatal dopaminergic depletion. In addition, the analysis regarding postmorbid neoplasia did not show any influence on correlation between striatal dopamine and motor deficit (data not shown). Finally, standardized quantitative analyses of this study minimized image noise and sampling errors resulting from visual or semiquantitative methods.

This study also had several limitations. First, the number of patients with neoplasia was relatively small compared to those without neoplasia. Second, the dopaminergic imaging has a flooring effect after 50% of neurons are lost^24^. Therefore, the correlation between the imaging parameters and clinical severities can be biased, especially in patients with severe motor handicap. Third, several patients with PD in this study had considerably lower UPDRS part III score compared to previously published studies and partly in the ranges of values reported for prodromal or preclinical PD^25,26^. Likewise, elderly subjects with comorbid diseases could have elevated UPDRS scores^27^. In fact, low UPDRS III values with relatively preserved ^18^F-FP-CIT uptake in patients with cancer might even represent motor difficulties due to the comorbid disease. However in this study, we performed brain MRI and ^18^F-FP-CIT-PET, and quantitative analysis of SUVR values compared with control group, and could minimize the clinical misdiagnosis. Fourth, we did not consider the other environmental/behavioral factors such as cigarette smoking that may linked to cancer, but protective against PD. Those who smoke are more likely to have smoking-related cancer. Most patients with PD are less likely to smoke, and many studies consistently suggested that smoking has a lowering effect on PD risk^28^. In PD patients, current smoking might protest dopamine neuronal degeneration in the striatum^29^. In this study of subjects, the smoking status was not different among groups, and therefore, we can presume the complex interaction among cigarette smoking, cancer and PD could be minimized. Although the epidemiological association among cigarette smoking, cancer and PD is puzzling, the verification of this complex association needs “two or more steps” multiple testing away from the epidemiological evidences. Finally, we did not perform follow-up evaluation about motor deficits, serial analysis of striatal dopamine uptake, or levodopa dosage.

In conclusion, PD patients with premorbid cancer have relatively preserved motor reserve in the striatum, especially in the posterior putamen. This finding provides imaging evidence that history of cancer has a protective effect on PD. The effects of premorbid cancer on progression of motor symptoms and striatal dopamine activity should be determined in future long-term follow-up studies.

## Methods

The study protocol was approved by the Institutional Review Board at Seoul St. Mary’s Hospital, and all subjects provided written informed consent to participate. All experiments were performed in accordance with relevant guidelines and regulations. The study is registered (Identification Number: KCT0005552, KCT0006293) at the Clinical Research Information Service (CRIS; http://cris.nih.go.kr), which is an online clinical trial registration system established by the Korea Centers for Disease Control and Prevention (KCDC) with support from the Korea Ministry of Health and Welfare (KMOHW), and is affiliated with the Primary Registries in the World Health Organization (WHO) Registry Network.

### Participants

Consecutive patients with newly diagnosed PD who visited the movement disorder clinic in a university-affiliated hospital between May 2018 and May 2020 were enrolled. PD was diagnosed based on the UK Parkinson's Disease Society Brain Bank clinical diagnostic criteria and Movement Disorder Society clinical diagnostic criteria for PD^30,31^. Twenty-two healthy subjects without any notable neurological or psychiatric diseases, and without previous history of benign tumor or cancer were recruited and included as controls (mean age = 69.0 ± 3.0 years, 11 male). Patient demographics of age, sex, disease duration, medical histories of hypertension, diabetes mellitus, and smoking status were collected. Motor symptoms were evaluated using the Unified Parkinson's Disease Rating Scale (UPDRS) and modified Hoehn and Yahr (H&Y) stage score^32,33^. All patients underwent brain magnetic resonance imaging (MRI) and positron emission tomography (PET) with ^18^F-N-(3-fluoropropyl)-2beta-carbon ethoxy-3beta-(4-iodophenyl) nortropane (^18^F-FP-CIT) at the time of diagnosis. All enrolled patients had decreased dopamine transporter uptake in the striatum (mainly in the posterior putamen) on visual analysis and the SUVR values of posterior putamen below the cut-off values of controls (cut-off value = 6.4712334).

All types of neoplasia (benign tumor and malignant cancer) were collected based on patient’s history and medical records. We divided patients into 3 groups of no prior neoplasia, premorbid precancerous condition (benign tumor), and premorbid malignant cancer. After enrollment, the patients were followed for a minimum of 8 months (mean ± SD; 20.9 ± 8.2 months) from the time of PD diagnosis.

Patients were excluded if they showed any of the following criteria: (1) normal dopamine transporter scan based on the Movement Disorder Society clinical diagnostic criteria for PD^31^, (2) atypical or secondary parkinsonism, (3) previous stroke or structural lesions on the basal ganglia, (4) use of anti-parkinsonian medications or other medications that influence striatal dopamine uptake, and (5) previous history of melanoma. In addition, subjects who developed benign tumors/cancers after PD diagnosis were excluded (n = 28). The mean duration of tumor development after PD diagnosis was 24.1 ± 8.4 months.

### PET imaging acquisition and processing

The ^18^F-FP-CIT-PET and computed tomography (CT) images were acquired using a Discovery STE PET/CT scanner (GE Healthcare, Milwaukee, WI, USA). At 3 h after the intravenous injection of an average of 3.7 MBq/kg of ^18^F-FP-CIT, brain CT scans were acquired for attenuation correction, followed by a 10-min ^18^F-FP-CIT emission PET scan. PET images were reconstructed in a 512 × 512 × 110 matrix with an ordered-subsets expectation maximization algorithm. The voxel size was 0.668 × 0.668 × 2 mm. Axial T1-weighted brain MRI with 3D-spoiled gradient-recalled sequences (512 × 512 matrix, voxel spacing 0.469 × 0.469 × 1 mm) were acquired using a 3.0-T scanner (MAGNETOM Verio, Siemens, Erlangen, Germany)^34,35^.

Statistical Parametric Mapping 8 software (SPM8; Wellcome Trust Centre for Neuroimaging, London, UK) implemented in MATLAB 2015a (MathWorks, Natick, MA, USA) was used for co-registration and spatial normalization of images and voxel-based comparisons. To spatially normalize ^18^F-FP-CIT PET images, an MR-guided conventional spatial normalization method was used^36,37^. PET images were co-registered to individual MR images and spatially normalized to the Montreal Neurological Institute space with the parameter normalizing, skull-stripped MR^36,37^. Volume of interest (VOI) templates of striatal subregions were obtained after subcortical parcellation and partial volume correction using FreeSurfer 5.1 (Massachusetts General Hospital, Harvard Medical School, Boston, MA; http://surfer.nmr.mgh.harvard.edu). The VOI templates of five striatal subregions and the cerebellum were normalized spatially to the MR template, and then subregional uptake values of average of both sides of caudate, putamen, globus pallidus, thalamus, ventral striatum, and cerebellum were calculated using in-house MATLAB 2015a programs, which were used in our previous studies^34,35^. Mean standardized uptake value ratio (SUVR) was calculated as target SUV divided by cerebellar SUV.

### Statistical analysis

Statistical analyses were performed using SPSS software version 24.0 for Mac (IBM Corporation, New York, NY, USA). Descriptive analyses and the Pearson's χ^2^ test and one-way analysis of variance (ANOVA) with least significant difference post-hoc test, Kruskal–Wallis test, or analysis of covariance (ANCOVA) were performed as appropriate. Correlations between motor deficits and SUVRs of each striatal subregion were determined using Pearson’s and Spearman’s correlation tests. To compare the differences of correlation coefficients between groups, Fisher’s z test was adopted. The ratio of SUVR of each striatal subregion/UPDRS part III score was also calculated. Higher values of this ratio mean preserved motor reserve. General linear models were used to calculate the difference in UPDRS part III score after controlling age, sex, symptom duration, hypertension, diabetes mellitus, smoking status, and SUVRs of posterior putamen. Statistical significance was set at *p* < 0.05.

## Supplementary Information

Supplementary Tables.

## Data Availability

Anonymized data generated during the current study are available from the corresponding author on reasonable request from individuals affiliated with research or health care institutions. The data are not publicly available due to their containing information that could compromise the privacy of the participants.
