# Author Correction: Premorbid cancer and motor reserve in patients with Parkinson’s disease

**DOI:** 10.1038/s41598-022-20204-9

**Published:** 2022-09-14

**Authors:** Yoon‑Sang Oh, Sang‑Won Yoo, Chul Hyoung Lyoo, Kwang‑Soo Lee, Joong‑Seok Kim

**Affiliations:** 1 Department of Neurology, College of Medicine, The Catholic University of Korea, Seoul, Republic of Korea; 2 Department of Neurology, Gangnam Severance Hospital, Yonsei University College of Medicine, Seoul, Republic of Korea; 3 Department of Neurology, Seoul St. Mary’s Hospital, College of Medicine, The Catholic University of Korea, 222, Banpo-daero, Seocho-gu, Seoul, 06591 Republic of Korea

Correction to: *Scientific Reports* 10.1038/s41598-022-13322-x, published online 03 June 2022

In the original version of this Article, Joong-Seok Kim was incorrectly affiliated with ‘Department of Neurology, College of Medicine, The Catholic University of Korea, Seoul St. Mary’s Hospital, 222, Banpo-daero, Seocho-gu, Seoul 06591, Republic of Korea.’ The correct affiliation is listed below:

Department of Neurology, Seoul St. Mary's Hospital, College of Medicine, The Catholic University of Korea, 222, Banpo-daero, Seocho-gu, Seoul, 06591, Republic of Korea.

Additionally, Affiliation 1 was incorrectly given as ‘Department of Neurology, College of Medicine, The Catholic University of Korea, Seoul St. Mary’s Hospital, 222, Banpo-daero, Seocho-gu, Seoul 06591, Republic of Korea.’ The correct affiliation is listed below:

Department of Neurology, College of Medicine, The Catholic University of Korea, Seoul, Republic of Korea.

The original Article has been corrected.

