# Supplementary material for: Premorbid cancer and motor reserve in patients with Parkinson’s disease
Source: Sci Rep. 2022 Jun 3;12:9254. doi: 10.1038/s41598-022-13322-x (PMC9166748; doi:10.1038/s41598-022-13322-x)
Supplement: Supplementary file 1 — Supplementary Tables. [file 41598_2022_13322_MOESM1_ESM.docx]

Supplementary Table 1. Comparisons of correlation coefficient between group without neoplasia and group with carcinoma by histopathological classification

|  |  |  | no neoplasia (group 1) vs. carcinoma (group 2) | | | |
| --- | --- | --- | --- | --- | --- | --- |
|  |  |  | Z1 | Z2 | Z | P |
| UPDRS part 3 | Caudate | | -0.335 | -0.375 | 0.229 | 0.409 |
|  |  | Anterior | -0.338 | -0.355 | 0.098 | 0.461 |
|  |  | Posterior | -0.268 | -0.231 | -0.216 | 0.415 |
|  | Putamen | | -0.231 | -0.653 | 2.446 | 0.007** |
|  |  | Anterior | -0.236 | -0.627 | 2.262 | 0.012* |
|  |  | Posterior | -0.188 | -0.522 | 1.931 | 0.027* |
|  | ` | Ventral | -0.194 | -0.628 | 2.513 | 0.006** |
|  | Globus pallidus | | 0.012 | -0.269 | 1.629 | 0.052 |
|  | Thalamus | | -0.270 | -0.300 | 0.226 | 0.410 |
|  | Ventral striatum | | -0.277 | -0.715 | 2.538 | 0.016* |

Analyses were performed using Pearson’s z test as following formula.

Z_a_ = 0.5[ln(1+*r* _a_) – (ln(1+ *r* _a_)]

$$Z= \frac{Z_{a}-Z_{b}}{\sigma_{Z_{a}-Z_{b}}}$$

$$\sigma_{Z_{a}-Z_{b}}=\sqrt{\frac{1}{n_{a}-3}+\frac{1}{n_{b}-3}}$$

Z _b_ = 0.5[ln(1+*r* _b_) – (ln(1+ *r*_b_)]

* < 0.05, ** < 0.01

Supplementary Table 2. UPDRS motor score estimates of the premorbid carcinoma group compared to the group without neoplasia

|  | Unadjusted | |  | Adjusted^a^ | |
| --- | --- | --- | --- | --- | --- |
|  | B (S.E.) | P |  | B (S.E.) | P |
| SUVRs of posterior putamen | -1.832 (0.417) | < 0.001 |  | -2.057 (0.412) | < 0.001 |
| No neoplasia | Reference |  |  | Reference |  |
| Premorbid carcinoma | -2.966 (1.424) | 0.038 |  | -3.870 (1.388) | 0.006 |

Analyses were performed by general linear model.

B, estimated difference; S.E. standard error, SUVRs, standardized uptake value ratios.

^a^ Adjusted for age, sex, symptom duration, hypertension, diabetes mellitus and smoking status.
